# Phase I trial of TAK‐385 in hormone treatment‐naïve Japanese patients with nonmetastatic prostate cancer

**DOI:** 10.1002/cam4.2442

**Published:** 2019-08-19

**Authors:** Hiroyoshi Suzuki, Hiroji Uemura, Atsushi Mizokami, Narihiko Hayashi, Yasuhide Miyoshi, Satoshi Nagamori, Yutaka Enomoto, Hideyuki Akaza, Takayuki Asato, Tadayuki Kitagawa, Kazuhiro Suzuki

**Affiliations:** ^1^ Department of Urology Toho University Sakura Medical Center Chiba Japan; ^2^ Department of Urology and Renal Transplantation Yokohama City University Medical Center Yokohama Japan; ^3^ Department of Integrative Cancer Therapy and Urology Graduate School of Medical Sciences Kanazawa University Kanazawa Japan; ^4^ Department of Urology Public University Corporation Yokohama City University Hospital Yokohama Japan; ^5^ Department of Urology Incorporated Administrative Agency National Hospital Organization Hokkaido Cancer Center Sapporo Japan; ^6^ Department of Urology Mitsui Memorial Hospital Tokyo Japan; ^7^ Department of Strategic Investigation on Comprehensive Cancer Network Interfaculty Initiative in Information Studies/Graduate School of Interdisciplinary Information Studies The University of Tokyo Tokyo Japan; ^8^ Oncology Clinical Research Department Oncology Therapeutic Area Unit for Japan and Asia Takeda Pharmaceutical Company Limited Osaka Japan; ^9^ Japan Development Center Takeda Pharmaceutical Company Limited Osaka Japan; ^10^ Department of Urology Graduate School of Medicine National University Corporation Gunma University Maebashi Japan

**Keywords:** hormone therapy, prostate cancer

## Abstract

This open‐label, phase I dose‐finding study evaluated the gonadotropin‐releasing hormone antagonist, TAK‐385, in Japanese patients with nonmetastatic prostate cancer. In a two‐part design, patients received daily oral TAK‐385 at doses of 320 (loading, day 1)/80 (maintenance, day 2 and thereafter), 320/120, 320/160, or 360/120 mg for 28 days in a dose‐escalation phase (part A, n = 13), and at 320/80 or 320/120 mg for up to 96 weeks in a randomized expansion phase (part B, n = 30). Primary endpoint in both parts was safety, including dose‐limiting toxicity in part A. Secondary endpoints included pharmacokinetics, pharmacodynamics, and prostate‐specific antigen concentration. Ten (77%) patients in part A and all patients in part B experienced an adverse event; hot flush (part A, n = 4; part B, n = 15), viral upper respiratory tract infection (part A, n = 1; part B, n = 10), and diarrhea (part B, n = 8) were most frequent. No dose‐limiting toxicities were observed (part A). In 12 evaluable patients (part A), TAK‐385 was rapidly absorbed after a single loading dose; on day 28 (maintenance dose), median steady‐state *T*_max_ was ~1‐2 hours and mean *t*_1/2z_ was 67‐79 hours. All doses rapidly reduced testosterone concentrations to castration levels within 1 week. Durable reductions in prostate‐specific antigen of >90% from baseline were observed through 96 weeks. TAK‐385 appeared tolerable and resulted in sustained reductions in testosterone to castration levels at all doses. The lowest loading/maintenance dose required for a clinical effect was 320/80 mg.

http://ClinicalTrials.gov: NCT02141659.

## Graphical abstract

TAK‐385 is an orally active gonadotropin‐releasing hormone (GnRH) antagonist with rapid testosterone‐lowering effects in clinical studies, achieving effective and sustained castration in patients with prostate cancer without the initial testosterone surge observed with GnRH agonists. To date, clinical trials of TAK‐385 for the treatment of prostate cancer have been conducted in a predominantly Western patient population. Building on these data, the current study establishes an appropriate dose range for TAK‐385 in Japanese patients with non‐metastatic prostate cancer, and evaluates safety, pharmacokinetics, pharmacodynamics and preliminary efficacy in this population.

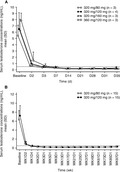

## 1 INTRODUCTION

In Japan, prostate cancer is the sixth leading cause of cancer‐related death among men.^1^ Although the incidence of prostate cancer in Japan is lower compared with that in Western countries,^2^ it has increased markedly over the past four decades, from 3944 cases recorded in 1980 to 86 100 cases in 2017.^1^ The prevalence of prostate cancer increases with age,^3^ and thus, with a growing elderly population in Japan,^4^ the need for effective and tolerable treatment options is rising.

Hormone therapy, either by surgery or drug therapy (eg, gonadotropin‐releasing hormone [GnRH] agonists or antagonists), is the most common treatment for localized, advanced, and metastatic prostate cancer.^5^, ^6^ Both GnRH agonists, such as leuprorelin acetate and goserelin acetate,^7^, ^8^ and the GnRH antagonist, degarelix,^9^ are approved in Japan. GnRH agonists result in androgen deprivation by overstimulating pituitary GnRH receptors.^10^, ^11^ With continued exposure, this effect leads to downregulation of GnRH receptors and a subsequent reduction in testosterone production 3‐4 weeks after treatment initiation.^10^, ^11^ However, an initial, transient rise in testosterone production (known as a testosterone flare) can lead to a temporary worsening of symptoms^12^, ^13^ and may require concomitant antiandrogen therapy.^13^ In contrast, the GnRH antagonist degarelix—a synthetic peptide derivative of natural GnRH^9^—has a more rapid onset of efficacy without an associated testosterone surge.^14^ However, peptide formulations are administered by subcutaneous injection, which can lead to injection site reactions.^15^ Therefore, research efforts are ongoing to develop convenient prostate cancer therapies with good clinical efficacy and tolerability.

TAK‐385 is an investigational, nonpeptide, orally active GnRH antagonist that is being evaluated as a novel therapeutic intervention for prostate cancer. TAK‐385 binds with high affinity to GnRH receptors in the anterior pituitary gland to suppress the hypothalamic‐pituitary‐gonadal axis.^16^, ^17^ There are currently only limited published data available on TAK‐385. A phase I study conducted in healthy male volunteers in the UK showed that oral administration of TAK‐385 40‐180 mg per day was tolerable and resulted in rapid decreases in serum testosterone concentration.^18^ Two completed phase II studies of TAK‐385 in patients in North America with intermediate‐risk or advanced prostate cancer revealed that treatment with oral TAK‐385 80 or 120 mg per day (with a 320 mg loading dose administered on day 1 in one of the trials) resulted in reductions in serum testosterone concentration to castration levels (<0.5 ng/mL) after week 1, and in prostate‐specific antigen (PSA) by >90% by the end of the 24‐week treatment period.^19^, ^20^

Data from the Japanese and American premenopausal healthy women have shown no major differences in the clinical effects of TAK‐385 between the two ethnic populations (http://ClinicalTrials.gov: NCT02792062 and NCT02093390; Takeda, study results disclosed on the http://ClinicalTrials.gov website). However, clinical data on the effects of TAK‐385 in men have, to date, been collected in a predominantly Western patient population. Given that ethnicity may impact on a drug's pharmacologic properties and effects (and may necessitate a different dosing regimen),^21^ this two‐part phase I study was designed to examine the tolerability, safety (including assessment of dose‐limiting toxicities [DLT]), pharmacokinetics (PK), pharmacodynamics (PD), and preliminary efficacy profile of TAK‐385 in Japanese men with nonmetastatic prostate cancer.

## 2 MATERIALS AND METHODS

### 2.1 Study design

**Figure 1:**
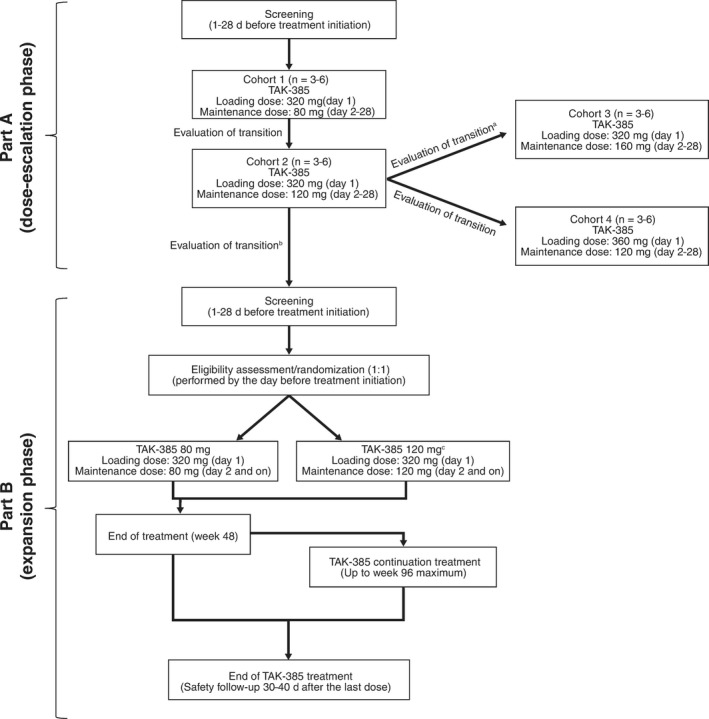
Study design. ^a^If tolerability was not confirmed in cohort 2, then a cohort was to be added, in which subjects were to receive a loading dose of 320 mg and a maintenance dose of 40 mg. ^b^If a cohort was added, and the additional cohort continued to the end and no problems with tolerability were found, then the study was to proceed to part B. ^c^If a cohort was added in part A, then in part B, the 120 mg group was to be replaced with a 40 mg group, in which patients were to receive a loading dose of 320 mg and a maintenance dose of 40 mg

This was a multicenter, open‐label, phase I, dose range‐finding study of TAK‐385 in Japanese adult men with nonmetastatic prostate cancer (http://ClinicalTrials.gov: NCT02141659). The two‐part design consisted of a dose‐escalation phase (part A) and an expansion phase (part B) (Figure 1).

Part A employed a 3+3 dose‐escalation schema. Enrollment to a new cohort proceeded if 0/3 or 1/6 patients reported a DLT in the previous cohort, with the exception that enrollment to cohorts 3 and 4 could proceed on the basis of confirmed tolerability of TAK‐385 in cohort 2. DLTs were assessed during a 28‐day treatment cycle and were defined as: any grade ≥3 adverse event (AE), based on the National Cancer Institute's Common Terminology Criteria for Adverse Events (NCI‐CTCAE) version 4.03, including clinical laboratory abnormalities (if they occurred at two consecutive time points ≥7 days apart, or at two time points ≥2 days apart for alanine aminotransferase [ALT] or aspartate aminotransferase [AST] >3 × the upper limit of normal [ULN], total bilirubin >2 × ULN, or prothrombin time‐international normalized ratio [PR‐INR] >1.5); or abnormal electrocardiogram (ECG) findings (QT/Fridericia‐corrected QT [QTcF] interval >500 ms after treatment initiation or a QT/QTcF interval prolongation of >0.60 ms post‐dose) at two time points ≥7 days apart.

Patients in part A received a single oral loading dose of TAK‐385 on day 1, followed by a daily oral maintenance dose on days 2‐28 in the following four loading/maintenance dose cohorts: 320/80 mg in cohort 1; 320/120 mg in cohort 2; 320/160 mg in cohort 3; and 360/120 mg in cohort 4. Doses were administered at least 30 minutes before breakfast. The loading/maintenance doses were selected based on data from studies previously conducted in Europe and North America.^18^, ^19^, ^20^ Cohorts 3 and 4 were included specifically to evaluate the tolerability of the 160‐mg maintenance dose and 360‐mg loading dose, respectively, which reflect the maximum tolerated maintenance and loading doses investigated in the Western studies. Patients in part A who completed the 28‐day treatment period were required to undergo a 1‐week, treatment‐free observation period before being switched to a GnRH agonist (eg, leuprorelin acetate) or alternative GnRH antagonist (eg, degarelix). Patients who discontinued TAK‐385 before the end of the 28‐day treatment period did not require this observation period.

Upon the successful completion of cohorts 1 and 2 in part A, a distinct group of patients were enrolled in the part B expansion phase and randomized 1:1 to receive daily TAK‐385 at a loading dose of 320 mg on day 1 and a maintenance dose of either 80 mg (320/80 mg group, cohort 1 dose) or 120 mg (320/120 mg group, cohort 2 dose) on day 2 until the end of treatment at week 48. At week 48, patients could either continue to receive TAK‐385 for a further 48 weeks (maximum total duration, 96 weeks) or be switched to a GnRH agonist or alternative GnRH antagonist, starting from the day after the final dose of TAK‐385.

### 2.2 Outcomes

Safety was the primary endpoint in parts A and B, determined via DLTs (part A only), AEs, vital signs, clinical laboratory tests, and 12‐lead ECGs. Secondary endpoints in parts A and B were the PK of unchanged TAK‐385 in plasma and PD as assessed by serum testosterone concentrations. Efficacy in terms of change in PSA concentration was a secondary endpoint in part B and an additional endpoint in part A. Other additional endpoints were, in both parts A and B, PD as assessed by luteinizing hormone (LH), follicle‐stimulating hormone (FSH), dihydrotestosterone (DHT), and sex hormone‐binding globulin (SHBG) concentrations, and, in part B only disease progression assessed by PSA or by imaging, and bone density.

### 2.3 Patients

Eligible patients were males aged ≥20 years with a pathologically confirmed diagnosis of prostate cancer and TNM classification of T1‐4 N0 M0 (or Tx N0 M0 for patients who had undergone radical prostatectomy). Patients were also required to have: an Eastern Cooperative Oncology Group performance status 0 or 1; a serum testosterone level at screening of >1.5 ng/mL; and a PSA level of >4.0 ng/mL in those with untreated prostate cancer or >0.2 ng/mL in those who had undergone prostatectomy or high‐intensity focused ultrasound therapy and/or radiotherapy. None of the patients had received prior hormone therapy for prostate cancer. Patients were excluded if they had received: ^125^I‐brachytherapy; 5‐alpha reductase inhibitors (except for patients who had been treated for male‐pattern alopecia); radiotherapy or prostatectomy within 16 weeks before the start of study; or chemotherapy to treat prostate cancer. Additional exclusion criteria included: uncontrolled diabetes or hypertension; heart disease, including myocardial infarction; clinically relevant ECG abnormalities; and clinical laboratory abnormalities, including serum creatinine ≥2.0 mg/dL, ALT or AST ≥ 1.5 × ULN, total bilirubin ≥ 2 × ULN, neutrophil count < 1500/mm^3^, platelet count <100 000/μL, and hemoglobin <10.0 g/dL.

This study was conducted in compliance with the Institutional Review Board regulations stated in the Good Clinical Practice regulations and guidelines, the Declaration of Helsinki, and all applicable local regulations. All patients provided written, informed consent.

### 2.4 Assessments

AEs were coded according to the Medical Dictionary for Regulatory Activities (MedDRA) version 20.0 and graded according to NCI‐CTCAE version 4.03. Treatment‐emergent AEs were defined as events that occurred after the start of study drug administration. Blood and urine samples for clinical laboratory tests (hematology, blood chemistry, metabolic panel, and urinalysis) were collected following >10 hours of fasting according to a predefined schedule. 12‐lead resting ECG recordings were assessed by investigators at regular intervals throughout the study. To determine the QTc value, Bazett's formula (QT/RR^0.5^) and Fridericia's formula (QT/RR^0.33^) were used to calculate the Bazett‐corrected QT (QTcB) and QTcF, respectively. Patients were assessed to determine if they had an observed QTcF of ≥500 ms, or ≥450 ms with a change from baseline of ≥30 ms.

In part B, bone mineral density (BMD) was determined using dual‐energy X‐ray absorptiometry at screening, and on weeks 25, 49, 73, and 97, or on study withdrawal; measurement sites were lumbar 2‐4 (L2‐4) and hip, which included femoral neck, trochanteric and intertrochanteric sites, and Ward's triangle.

In part A, blood samples for PK analyses were collected at the following time points: pre‐dose on days 1‐3, 7, 12‐15, 21, 28‐31, and 35; at 0.5, 1, 1.5, 2, 4, 6, 8, and 12 hours post‐dose on days 1 and 14; 2 hours post‐dose on days 3 and 7; and 1, 2, 4, 8, and 12 hours post‐dose on day 28. In part B, blood samples for PK analyses were collected: pre‐dose on days 1, 2, and 4 of week 1 and on day 1 of weeks 2, 3, 5, 9, 13, 17, 21, 25, 37, and 49; and 2 hours post‐dose on day 1 of weeks 1 and 5.

In part A, blood samples for measurement of serum testosterone, LH, FSH, DHT, and SHBG concentrations were collected at screening, and on days 2, 3, 7, 14, 21, 28, 31, and 35 for part A. In part B, blood samples were collected at screening, on days 1, 2, and 4 of week 1, and on day 1 of weeks 2‐5, 9, 13, 17, 21, 25, 37, and 49. Castration rate was defined as the proportion of patients with serum testosterone <0.5 ng/mL at all visits through day 1 of week 5 to day 1 of week 25.

Tumor progression was determined by changes in PSA concentration per the Prostate Cancer Handling Guidelines, Fourth Edition.^22^ Progression of disease was defined as an increase of ≥25% in the PSA value measured at least 4 weeks after the nadir, or at least 12 weeks after the nadir with an absolute increase of ≥2 ng/mL if the nadir value had been recorded during screening. Computed tomography (CT) or magnetic resonance imaging of thoracic, abdominal, and pelvic regions, and bone scintigraphy was employed to verify the presence or absence of new lesions. In part A, imaging was performed at screening and at any point as appropriate during treatment. In part B, imaging was performed at screening (if the patient had not undergone imaging within 28 days prior to treatment initiation), and on day 1 of weeks 49 and 97, or at study withdrawal.

### 2.5 Statistical analysis

TAK‐385 plasma PK parameters were calculated using noncompartmental methods. Graphical assessments of dose‐proportionality of maximum concentration (*C*_max_) and area under the plasma concentration‐time curve (AUC) during a dosing interval at steady state (*C*_max,ss_ and AUC_τ,ss_) were conducted on days 14 and 28 by plotting individual dose‐normalized exposure parameters vs dose (*C*_max,ss_/D and AUC_τ,ss_/D). A power regression analysis was also performed for each parameter (*C*_max,ss_/D and AUC_τ,ss_/D) using the power model.

In part B, 15 patients per group were established as the number required for an approximately 80% chance of detecting AEs characterized by a true incidence rate of 10%.

All data analyses were performed using SAS version 9.2 (SAS Institute Inc, Cary, NC). Descriptive statistics were calculated for all endpoints.

## 3 RESULTS

### 3.1 Patient disposition and treatment exposure

In part A, 13 patients were enrolled and treated (Table 1). One patient in cohort 2 discontinued study treatment prematurely after receiving the 320‐mg loading dose due to a major protocol deviation; the remaining 12 (92%) patients completed the 28‐day treatment period and safety follow‐up.

**Table 1:** Patient demographics and disease characteristics at baseline

	Part A					Part B		
	Cohort 1 (320/80 mg) (n = 3)	Cohort 2 (320/120 mg) (n = 4)	Cohort 3 (320/160 mg) (n = 3)	Cohort 4 (360/120 mg) (n = 3)	Total (N = 13)	320/80 mg (n = 15)	320/120 mg (n = 15)	Total (N = 30)
Mean age, years (SD)	73.3 (5.51)	72.0 (5.60)	75.0 (6.08)	71.7 (3.51)	72.9 (4.79)	74.5 (4.84)	74.4 (5.53)	74.5 (5.10)
Mean testosterone, ng/mL (SD)	5.44 (1.68)	6.51 (2.62)	6.32 (1.75)	7.85 (1.19)	6.53 (1.92)	6.37 (1.99)	7.24 (2.24)	6.81 (2.13)
Mean PSA, ng/mL (SD)	6.73 (6.38)	12.13 (5.30)	8.46 (2.33)	6.41 (5.79)	8.72 (5.15)	10.60 (14.56)	6.63 (6.66)	8.61 (11.31)
ECOG performance status 0, n (%)	3 (100)	4 (100)	3 (100)	3 (100)	13 (100)	15 (100)	15 (100)	30 (100)
Mean disease duration, weeks (SD)	74.3 (118.0)	29.3 (43.9)	7.0 (0.2)	168.1 (280.8)	66.6 (141.0)	139.0 (195.0)	146.7 (185.8)	142.8 (187.2)
Gleason score, n (%)								
6	0	0	0	0	0	5 (33.3)	5 (33.3)	10 (33.3)
7	2 (66.7)	3 (75.0)	2 (66.7)	3 (100)	10 (76.9)	6 (40.0)	8 (53.3)	14 (46.7)
8	0	1 (25.0)	1 (33.3)	0	2 (15.4)	3 (20.0)	0	3 (10.0)
9	1 (33.3)	0	0	0	1 (7.7)	1 (6.7)	2 (13.3)	3 (10.0)
TNM classification, n (%)								
T1	1 (33.3)	2 (50.0)	1 (33.3)	0	4 (30.8)	7 (46.7)	3 (20.0)	10 (33.3)
T2	1 (33.3)	1 (25.0)	1 (33.3)	1 (33.3)	4 (30.8)	2 (13.3)	5 (33.3)	7 (23.3)
T3	0	1 (25.0)	1 (33.3)	1 (33.3)	3 (23.1)	1 (6.7)	1 (6.7)	2 (6.7)
TX	1 (33.3)	0	0	1 (33.3)	2 (15.4)	5 (33.3)	6 (40.0)	11 (36.7)

Abbreviations: ECOG, Eastern Cooperative Oncology Group; PSA, prostate‐specific antigen; SD, standard deviation; TNM, primary tumor‐regional lymph nodes‐distant metastasis.

After assessing the safety of TAK‐385 as satisfactory in cohorts 1 and 2, 30 patients were enrolled in part B (Table 1). Mean treatment durations were 77.5 weeks (standard deviation [SD], 31.6) for the 320/80 mg group and 71.8 weeks (SD, 29.4) for the 320/120 mg group. Overall, 26 (87%) patients, 13 from each group, completed the initial 48‐week study period. The four (13%) patients in part B who discontinued TAK‐385 treatment before the end of week 48 did so due to drug‐related AEs (320/80 mg group [n = 2]; 320/120 mg group [n = 1]) or lack of efficacy (320/120 mg group [n = 1]). Twenty patients (320/80 mg group: n = 11; 320/120 mg group: n = 9) extended TAK‐385 treatment for up to another 48 weeks. Ten patients in the 320/80 mg group and eight in the 320/120 mg group completed 96 weeks’ therapy.

### 3.2 Safety

No DLTs were reported during part A. Across all cohorts, 10/13 (77%) patients experienced an AE; all of these AEs were grade 1 or 2 in intensity except for one report of grade 3 hypertension (observed in one patient in cohort 1), which was not considered to be drug‐related and thus was not classified as a DLT. The most common all‐cause and drug‐related AEs are summarized in Table S1. No serious AEs, AEs leading to study drug discontinuation, or deaths were observed during part A.

All patients in part B reported at least one AE; in most patients, AEs were categorized as grade 1 or 2 (n = 10 [67%] for both the 320/80 and 320/120 mg groups). The most common all‐cause and drug‐related AEs by preferred term are presented in Table 2. Five patients from each treatment group experienced grade ≥3 AEs. Hypertension was the only grade ≥3 AE reported in more than one patient overall; the two patients with grade ≥3 hypertension were both in the 320/80 mg group. Three patients from each group had serious AEs. Cerebral infarction, which was reported in one patient in the 320/80 mg group, was the only serious AE to be considered drug‐related; the patient who developed this AE had preexisting (controlled) hypertension, dyslipidemia, and hyperglycemia. The proportion of patients who discontinued the study drug due to AEs was 20% in the 320/80 mg group (n = 3; appendicitis perforated, cerebral infarction [drug‐related serious AE], and hypertension) and 13% in the 320/120 mg group (n = 2; arrhythmia and jaundice cholestatic). No deaths occurred during part B.

**Table 2:** Most frequent all‐cause (≥3 patients overall) and drug‐related AEs in the expansion phase (part B)

	TAK‐385 320/80 mg (n = 15)		TAK‐385 320/120 mg (n = 15)	
n (%)	All‐cause	Drug‐related	All‐cause	Drug‐related
Any AE	15 (100)	11 (73.3)	15 (100)	14 (93.3)
Hot flush	6 (40.0)	6 (40.0)	9 (60.0)	9 (60.0)
Viral upper respiratory tract infection	5 (33.3)	—	5 (33.3)	—
Diarrhea	3 (20.0)	—	5 (33.3)	—
Constipation	1 (6.7)	—	4 (26.7)	—
AST increased	1 (6.7)	—	3 (20.0)	1 (6.7)
ALT increased	1 (6.7)	—	2 (13.3)	2 (13.3)
Arthralgia	1 (6.7)	1 (6.7)	2 (13.3)	—
Blood cholesterol increased	1 (6.7)	1 (6.7)	2 (13.3)	2 (13.3)
Dental caries	1 (6.7)	—	2 (13.3)	—
Dysuria	1 (6.7)	—	2 (13.3)	—
Eczema	1 (6.7)	1 (6.7)	2 (13.3)	2 (13.3)
Gynecomastia	2 (13.3)	2 (13.3)	1 (6.7)	1 (6.7)
Headache	1 (6.7)	1 (6.7)	2 (13.3)	1 (6.7)
Hepatic function abnormal	2 (13.3)	2 (13.3)	1 (6.7)	1 (6.7)
Hypertension	2 (13.3)	2 (13.3)	1 (6.7)	1 (6.7)
Muscular weakness	2 (13.3)	1 (6.7)	1 (6.7)	1 (6.7)
Nausea	1 (6.7)	—	2 (13.3)	—
Vomiting	2 (13.3)	—	1 (6.7)	—
Weight increased	—	—	3 (20.0)	3 (20.0)

Abbreviations: AEs, adverse events; ALT, alanine aminotransferase; AST, aspartate aminotransferase.

Mean L2‐4, femoral neck, and total hip BMD values were numerically similar between the two part B dose groups at baseline and decreased following treatment. Mean femoral neck and total hip BMD appeared to decrease in a treatment duration‐dependent, but not dose‐dependent, way. At week 49, mean percent change from baseline in femoral neck and total hip BMD (320/80 vs 320/120 mg group) was −2.62% vs −1.19% and −2.27% vs −1.63%, respectively. At week 97, the respective mean percent changes were −4.87% vs −3.04% and −5.26% vs −5.54% (Figure S1). Mean decrease from baseline in L2‐4 BMD appeared to be neither treatment duration‐dependent nor dose‐dependent at week 49 (−4.12% vs −2.71%, respectively) and at week 97 (−4.01% vs −3.29%, respectively) (Figure S1).

Among all patients in part A and part B, vital signs and clinical laboratory test results showed no clinically significant changes from baseline (data not shown). In part A, no abnormal 12‐lead ECG readouts were observed and no ECG‐related AEs were reported. In part B, 2/15 (13%) patients in the 320/80 mg group and 5/15 (33%) patients in the 320/120 mg group experienced a QTcF interval prolongation. No patient had a QTcF interval of ≤50 ms. An abnormal 12‐lead ECG readout was reported in one (7%) patient each in the 320/80 mg group at week 73 and in the 320/120 mg group at week 3. AEs related to the ECGs were ECG QT prolonged and ECG ST segment elevation, which were reported in one patient each. Only the event of ECG QT prolonged was deemed drug‐related; no action was taken and the event resolved.

### 3.3 Pharmacokinetics

Data from 12 patients in part A who were PK‐evaluable were assessed for unchanged plasma TAK‐385. Mean plasma concentration‐time profiles for patients in the four cohorts on days 1, 14, and 28 are shown in Figure S2. In patients in cohorts 1‐3, who received a loading dose of 320 mg, the plasma concentration of unchanged TAK‐385 increased rapidly, with a median time to *C*_max_ (*T*_max_) ranging from 1.00 to 1.55 hours on day 1 (Table 3). On day 14, TAK‐385 systemic exposure (AUC_Τ,ss_) increased in a dose‐dependent way. In the power model, point estimates of the power index at day 14 were 1.756 (95% CI, –0.387, 3.899) for *C*_max,ss_/D and 0.680 (−0.919, 2.280) for AUC_Τ,ss_/D. On day 28, *C*_max,ss_/D was −0.709 (−3.477, 2.060) and AUC_Τ,ss_/D was −0.599 (−2.732, 1.535). Patients in cohort 4, who received a loading dose of 360 mg, also showed a rapid increase in the plasma concentration of TAK‐385, with a median *T*_max_ of 1.35 hours on day 1 (Table 3). At a maintenance dose of 120 mg, the systemic exposure of TAK‐385 in cohort 4 was similar to that seen in cohort 2 (Table 3).

**Table 3:** Summary of plasma pharmacokinetics of TAK‐385 in the dose‐escalation phase (part A)

	Cohort 1 (320/80 mg) (n = 3)	Cohort 2 (320/120 mg) (n = 3)	Cohort 3 (320/160 mg) (n = 3)	Cohort 4 (360/120 mg) (n = 3)
Day 1				
Mean AUC_τ_ (h.ng/mL) (SD), CV (%)	679.7 (96.34), 14.2	933.0 (604.30), 64.8	761.7 (249.78), 32.8	663.3 (501.85), 75.7
Mean *C*_max_ (ng/mL) (SD), CV (%)	191.7 (94.41), 49.3	198.0 (62.39), 31.5	250.0 (207.56), 83.0	254.0 (330.58), 130.1
Median *T*_max_ (h) (range), CV (%)	1.00 (0.47‐4.00), 104.5	1.55 (1.00‐1.95), 31.8	1.00 (0.45‐1.00), 38.9	1.35 (1.00‐1.92), 32.6
Day 14				
Mean AUC_last,ss_ (h.ng/mL) (SD), CV (%)	195.0 (47.15), 24.2	360.9 (289.27), 80.2	740.0 (387.83), 52.4	374.0 (195.54), 52.3
Mean AUC_τ,ss_ (h.ng/mL) (SD), CV (%)	199.0 (49.49), 24.9	363.8 (291.00), 80.0	741.7 (385.96), 52.0	379.0 (199.65), 52.7
Mean *C*_max,ss_ (ng/mL) (SD), CV (%)	23.5 (1.42), 6.0	94.4 (115.08), 121.9	216.1 (151.78), 70.2	65.6 (40.84), 62.3
Mean *C*_min,ss_ (ng/mL) (SD), CV (%)	4.8 (1.98), 41.4	8.5 (5.9), 69.4	12.2 (3.72), 30.5	9.7 (5.01), 51.6
Median *T*_max,ss_ (h) (range), CV (%)	0.58 (0.45‐1.00), 42.3	0.60 (0.33‐0.97), 50.3	0.55 (0.50‐1.00), 40.3	1.02 (0.57‐2.03), 62.1
Day 28				
Mean AUC_last,ss_ (h.ng/mL) (SD), CV (%)	629.0 (218.82), 34.8	817.3 (609.91), 74.6	1217 (1107.30), 91.0^a^	701.7 (406.51), 57.9
Mean AUC_τ,ss_ (h.ng/mL) (SD), CV (%)	239.0 (75.02), 31.4	323.4 (230.40), 71.2	469.0 (458.21), 97.7^a^	227.5 (138.37), 60.8
Mean *C*_max,ss_ (ng/mL) (SD), CV (%)	38.1 (21.74), 57.1	34.4 (24.96), 72.5	94.7 (114.98), 121.4^a^	25.56 (16.28), 63.7
Median T_max,ss_ (h) (range), CV (%)	2.12 (0.98‐4.00), 64.4	1.95 (1.00‐3.92), 65.0	1.04 (1.00‐1.08), 5.4^a^	2.03 (1.00‐2.05), 35.5
Mean CL/*F*_ss_ (L/h) (SD), CV (%)	359.7 (120.90), 33.6	637.3 (604.29), 94.8	656.0 (642.05), 97.9^a^	788.0 (663.01), 84.1
Mean *t*_1/2z_ (h) (SD), CV (%)	76.07 (11.19), 14.7	78.70 (20.70), 26.3	67.45 (8.70), 12.9^a^	67.10 (6.94), 10.3
Mean *V*_z_/*F*_ss_ (L) (SD), CV (%)	39 130 (13 492), 34.5	63 800 (46 682), 73.2	67 400 (70 145), 104.1^a^	77 070 (67 528), 87.6

Abbreviations: AUC, area under the concentration‐time curve; AUC_last,ss_, AUC from time 0 to time of the last quantifiable concentration, at steady state; AUC_τ_, AUC during a dosing interval; AUC_τ,ss_, AUC_τ_, at steady state; CL/F_ss_, apparent clearance after extravascular administration, at steady state, calculated using AUC_τ_; *C*_max_, maximum observed concentration; *C*_max,ss_, *C*_max_ during a dosing interval, at steady state; *C*_min,ss_, minimum observed concentration during a dosing interval, at steady state; CV, coefficient of variation; SD, standard deviation; *t*_1/2z_, terminal disposition phase half‐life; *T*_max_, time of first occurrence of maximum observed concentration; *T*_max,ss_, *T*_max_ at steady state; *V*_z_/*F*_ss_, apparent volume of distribution during the terminal disposition phase after extravascular administration, calculated using AUC_τ_.

a n = 2.

For patients in part B, mean plasma concentration‐time profiles for the 320/80 mg and 320/120 mg groups are shown in Figure S3. Mean concentration of unchanged TAK‐385 at the end of a dosing interval (*C*_trough_) increased in a dose‐proportional manner; *C*_trough_ was 3.972 ng/mL on day 2 of week 1 and 6.406 ng/mL on day 1 of week 49 with a maintenance dose of 80 mg, and 7.565 ng/mL, and 11.95 ng/mL, respectively, with a maintenance dose of 120 mg.

### 3.4 Pharmacodynamics

**Figure 2:**
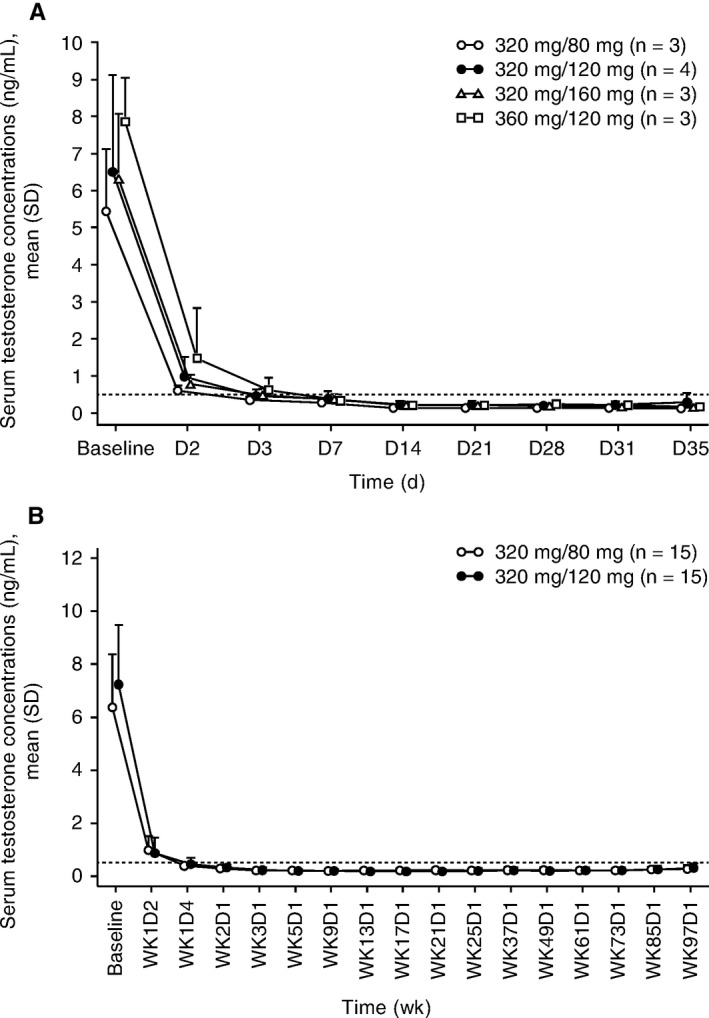
Mean (standard deviation) serum concentration‐time profiles of testosterone, (A) by dose cohort in the dose‐escalation phase (part A), and (B) by dose group in the expansion phase (part B). D, day; SD, standard deviation; WK, week

In part A, mean serum testosterone concentration decreased substantially within 2 days of starting TAK‐385 treatment in all cohorts. Mean concentration decreased to below the castration threshold level (0.5 ng/mL) by day 3 in cohorts 1‐3 and by day 7 in cohort 4, and remained below this level until day 35, 7 days after the last dose (Figure 2).

In part B, mean serum testosterone concentrations in both groups were reduced to below 0.5 ng/mL by day 4 of treatment (from mean baseline values of 6.37 ng/mL in the 320/80 mg group and 7.24 ng/mL in the 320/120 mg group), and remained suppressed at below castration levels for the duration of the study (Figure 2). Castration rates were 100% in both treatment groups. The testosterone concentration of one patient in the 320/120 mg group increased above the castration level at week 37 (0.56 ng/mL), but returned to below the castration level by week 49 (0.36 ng/mL).

Consistent and coincident with the decrease of serum testosterone concentration in patients in parts A and B, TAK‐385 treatment resulted in rapid decreases in serum LH (Figure S4), FSH (Figure S5), and DHT (data not shown) concentrations. However, the serum concentration of SHBG remained unchanged for the duration of treatment (data not shown).

### 3.5 Efficacy

In part A, mean PSA levels decreased across all cohorts. Mean change from baseline at day 28 ranged from −32.1% to −83.3% (Table S2).

**Figure 3:**
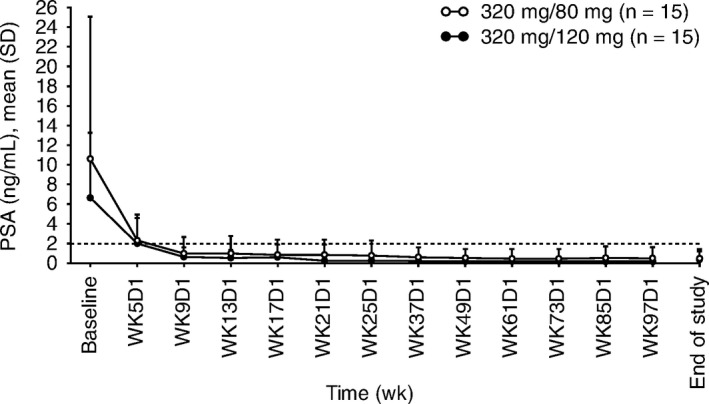
Mean (SD) concentration‐time profile of prostate‐specific antigen by dose group in the expansion phase (part B). D, day; PSA, prostate‐specific antigen; SD, standard deviation; WK, week

In part B, patients enrolled in the 320/80 mg and 320/120 mg groups had mean PSA values of 10.6 ng/mL (SD, 14.6) and 6.6 ng/mL (SD, 6.7), respectively, at baseline. Mean PSA concentrations declined steadily throughout the study. At the first assessment time point (week 5), mean change from baseline was −73.6% (SD, 20.2%) in the 320/80 mg group and –75.9% (SD, 17.06%) in the 320/120 mg group (Figure 3). PSA suppression was maintained until the end of the 96‐week treatment period; mean change from baseline at this time point was −95.1% (SD, 6.6%) in the 320/80 mg group and −93.6% (SD, 12.2%) in the 320/120 mg group. Only one patient (320/120 mg group) was determined to have disease progression per PSA changes (ie, had a ≥25% increase in PSA and an absolute increase of ≥2 ng/mL from the nadir, at week 17 [4.88 ng/mL] from a nadir at week 13 [2.46 ng/mL]); imaging analyses showed that no patients in either treatment group developed new lesions during the 96‐week study.

## 4 DISCUSSION

Previous studies in the UK and North America have suggested that TAK‐385 can provide clinically meaningful benefits in patients with prostate cancer.^18^, ^19^, ^20^ Therefore, we conducted this phase I study to examine the safety, tolerability, PK, PD, and efficacy of TAK‐385 in Japanese patients with nonmetastatic prostate cancer. Overall, TAK‐385 was well tolerated at all dose levels, with no DLTs reported (in part A) and most AEs being of grade 1 or 2 intensity. The PK analyses showed that TAK‐385 was rapidly absorbed with systemic exposure to the unchanged drug increasing in a dose‐proportional manner. Pharmacodynamically, treatment with TAK‐385 induced a rapid and sustained reduction in serum testosterone concentration to below castration level in this patient population. In part B, the preliminary efficacy of TAK‐385 was confirmed, with a mean reduction in serum PSA concentration of >90% by the end of the study in both dose groups.

In line with the currently established safety profile, no new safety concerns were identified for TAK‐385, and AEs reported in both part A and part B were generally mild or moderate. The most common AE reported during the study was hot flush. This finding is consistent with previous reports on TAK‐385 in Western patients^18^, ^19^, ^20^ and may be considered a class effect, as hot flush is also seen with degarelix.^10^ ECG irregularities (QTcF interval prolongation) and AEs related to ECGs were noted in a minority of patients in part B. We chose to specifically evaluate the cardiac effects of TAK‐385 because prolonged QTcF interval was reported in a prior phase I study of TAK‐385 in healthy subjects, and in a study of degarelix and leuprorelin acetate in patients with prostate cancer.^18^, ^23^ GnRH‐targeted agents may affect the QTcF interval due to their effects on serum testosterone concentration, as low levels of this hormone are associated with a prolonged QT interval.^24^

Previous studies have shown that use of androgen‐deprivation therapy, such as leuprorelin acetate and goserelin acetate, decreases bone density.^25^, ^26^ This is expected as gonadal hormones are essential for maintaining bone health. In this study, we found decreases from baseline in L2‐4, femoral neck, and total hip BMD during the course of TAK‐385 treatment, with mean decreases in BMD from baseline at week 49 ranging from −1.2% to −4.1%. The magnitude of BMD reduction is similar to previous reports, in which reductions from baseline of between −2.6% and −4.1% were reported in patients who received ≤6 or >6 months of androgen‐deprivation therapy, respectively.^25^, ^27^ Prevention and treatment of bone loss are therefore essential for patients receiving androgen‐deprivation therapy, such as TAK‐385.^28^

PK analysis showed that TAK‐385 was rapidly absorbed following oral dosing. In cohorts 1‐4 in part A, median T_max_ on day 1 ranged between 1.00 and 1.55 hours, and on day 14 ranged between 0.55 and 1.02 hours; these results are consistent with the mean *T*_max_ of 1.01 to 1.59 hours observed in Western healthy volunteers who received oral TAK‐385 20‐180 mg for 14 days.^18^ The point estimates of the power index in the power model for the relationship between dose‐normalized exposures (*C*_max_ and AUC) of unchanged TAK‐385 and dose were not always close to 0 on day 14 and day 28. However, the 95% CIs of all values included 0 and the *t*_1/2z_ on day 28 was similar in all cohorts, suggesting an absence of nonlinear PK on day 14 and day 28.

Both the escalation (part A) and expansion (part B) parts of the study demonstrated that TAK‐385 resulted in effective castration. Mean testosterone levels decreased to below the castration threshold of 0.5 ng/mL within 1 week of starting TAK‐385 in all treatment groups. Additionally, there was no evidence of testosterone flare, consistent with the mode of action of GnRH antagonists.^10^ The lowest TAK‐385 loading/maintenance dose tested for continuous castration was 320/80 mg. No notable differences in mean testosterone levels were observed beyond 1 week of treatment among the various loading/maintenance doses used, which is consistent with a previously published phase I study of TAK‐385 in healthy males.^18^ In addition to lowering testosterone concentration, TAK‐385 also decreased LH, FSH, and DHT levels. As LH and FSH form part of the hypothalamic‐pituitary‐gonadal axis, their suppression is expected, and these findings are consistent with a prior phase II study of TAK‐385 and a phase III study of degarelix and leuprolide.^10^, ^19^ Since DHT is converted from testosterone,^29^ the suppression of DHT by TAK‐385 is also expected. The concentration of SHBG, nevertheless, remained unchanged during the course of TAK‐385 treatment; SHBG is not part of the hypothalamic‐pituitary‐gonadal axis^30^, ^31^ and is therefore not targeted by GnRH antagonists, demonstrating the specificity of TAK‐385.

Because PSA expression is regulated by androgen receptors,^32^ the observed decrease in PSA concentration in response to TAK‐385‐induced castration was expected. In part A, notable reductions in PSA were seen by day 28. In part B, PSA decreased from baseline by >70% in the 320/80 mg and 320/120 mg groups after 4 weeks of TAK‐385 treatment; PSA suppression was then sustained throughout the full course of treatment. PSA reductions following oral TAK‐385 were also observed in a phase II study in North American patients with prostate cancer who received TAK‐385 at the same doses as used in the present study (320/80 mg or 320/120 mg). Patients in that trial had a >95% reduction from baseline in mean PSA concentration after 24 weeks of treatment.^20^ Furthermore, the effect of TAK‐385 on PSA is consistent with that seen with other GnRH agents, including leuprorelin acetate and degarelix.^19^, ^20^

A notable limitation of this study was the small number of patients. However, despite this shortcoming, the observed clinical effects of TAK‐385, including marked reductions in testosterone and PSA concentrations relative to baseline, were consistent with those reported in Western studies.^18^, ^19^, ^20^ Inclusion of a noncomparative, active control cohort, in which patients were treated with the GnRH antagonist degarelix might have been beneficial to provide further context for the results, especially the safety findings.

In summary, in Japanese patients with nonmetastatic prostate cancer, administration of TAK‐385 (at a 320 or 360 mg loading dose followed by 80‐160 mg maintenance dose) appeared tolerable for up to 96 weeks. Within the context of a small phase I trial, TAK‐385 resulted in long‐term suppression of serum testosterone concentrations to below castration levels, without testosterone flares, and in sustained reductions in serum PSA concentrations. In general, the findings reported for Japanese patients were similar to those seen in patients in Western studies. Based on the therapeutic benefits of TAK‐385 reported in this study and in non‐Japanese phase II studies, a randomized, global phase III study is ongoing to confirm the efficacy of TAK‐385 vs leuprolide acetate in men with advanced prostate cancer http://ClinicalTrials.gov: NCT03085095). If this trial is successful and TAK‐385 is approved, it will be interesting to evaluate how real‐world adherence to this once‐daily oral medication compares with that for injectable depot GnRH agents.

## 5 DATA SHARING STATEMENT

Takeda makes patient‐level, de‐identified datasets and associated documents available after applicable marketing approvals and commercial availability have been received, an opportunity for the primary publication of the research has been allowed, and other criteria have been met as set forth in Takeda's Data Sharing Policy (see http://www.TakedaClinicalTrials.com for details). To obtain access, researchers must submit a legitimate academic research proposal for adjudication by an independent review panel, who will review the scientific merit of the research and the requestor's qualifications and conflict of interest that can result in potential bias. Once approved, qualified researchers who sign a data sharing agreement are provided access to these data in a secure research environment.

## CONFLICT OF INTEREST

None declared.
