# Supplementary material for: Phase I trial of TAK‐385 in hormone treatment‐naïve Japanese patients with nonmetastatic prostate cancer
Source: Cancer Med. 2019 Aug 19;8(13):5891–902. doi: 10.1002/cam4.2442 (PMC6792482; doi:10.1002/cam4.2442)

**List of Supporting information**

**SUPPLEMENTARY TABLE S1** Most frequent all-cause (≥2 patients overall) and drug-related adverse events in the dose-escalation phase (Part A)

|  | **Cohort 1**  **320/80 mg**  **(n = 3)** | | **Cohort 2**  **320/120 mg**  **(n = 4)** | | **Cohort 3**  **320/160 mg**  **(n = 3)** | | **Cohort 4**  **360/120 mg**  **(n = 3)** | | **Total**  **(N = 13)** | |
| --- | --- | --- | --- | --- | --- | --- | --- | --- | --- | --- |
| **AE, n (%)** | **All cause** | **Drug-related** | **All cause** | **Drug-related** | **All cause** | **Drug-related** | **All cause** | **Drug-related** | **All  cause** | **Drug-related** |
| Hot flush | 2 (66.7) | 1 (33.3) | - | - | - | - | 2 (66.7) | 1 (33.3) | 4 (30.8) | 2 (15.4) |
| ALT increased | 1 (33.3) | - | 1 (25.0) | - | - | - | - | - | 2 (15.4) | - |
| Constipation | 2 (66.7) | - | - | - | - | - | - | - | 2 (15.4) | - |
| Hepatic function abnormal | - | - | - | - | 1 (33.3) | 1 (33.3) | 1 (33.3) | 1 (33.3) | 2 (15.4) | 2 (15.4) |
| Musculoskeletal pain | - | - | 1 (25.0) | - | 1 (33.3) | - | - | - | 2 (15.4) | - |

AE, adverse event; ALT, alanine aminotransferase.

**SUPPLEMENTARY TABLE S2** Prostate-specific antigen concentration at baseline and on day 28, and percent change from baseline on day 28, by cohort in the dose-escalation phase (Part A)

|  |  |  | **PSA concentration (ng/mL)** | | **Change from baseline in PSA (%)** | |
| --- | --- | --- | --- | --- | --- | --- |
|  | **Treatment** | **n** | **Mean (SD)** | **95% CI** | **Mean (SD)** | **95% CI** |
| Baseline | Cohort 1 | 3 | 6.73 (6.38) | –9.13, 22.58 |  |  |
|  | Cohort 2 | 4 | 12.13 (5.30) | 3.71, 20.56 |  |  |
|  | Cohort 3 | 3 | 8.46 (2.33) | 2.68, 14.24 |  |  |
|  | Cohort 4 | 3 | 6.41 (5.79) | –7.98, 20.80 |  |  |
| D28 | Cohort 1 | 3 | 1.80 (1.54) | –2.03, 5.62 | –76.37 (16.13) | –116.44, –36.30 |
|  | Cohort 2 | 4 | 4.18 (2.65) | –0.03, 8.39 | –60.10 (32.58) | –111.94, –8.27 |
|  | Cohort 3 | 3 | 4.93 (2.41) | –1.06, 10.92 | –32.10 (53.43) | –164.83, 100.63 |
|  | Cohort 4 | 3 | 1.10 (1.02) | –1.44, 3.63 | –83.33 (0.75) | –85.20, –81.47 |

CI, confidence interval; D, day; PSA, prostate-specific antigen; SD, standard deviation.

**SUPPLEMENTARY FIGURE S1** Mean (standard deviation) percent change from baseline in bone mineral density in the expansion phase (Part B): (A) L2–4, (B) femoral neck, and (C) total hip. BMD, bone mineral density; D, Day; SD, standard deviation; WK, week.


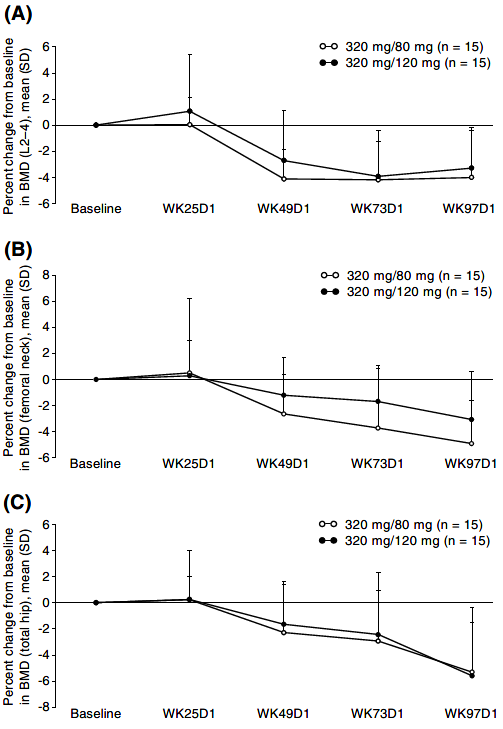


**SUPPLEMENTARY FIGURE S2** Mean (standard deviation) plasma concentration–time profiles of TAK-385 by dose cohort in the dose-escalation phase (Part A) on (A) day 1, (B) day 14, and (C) day 28. SD, standard deviation.


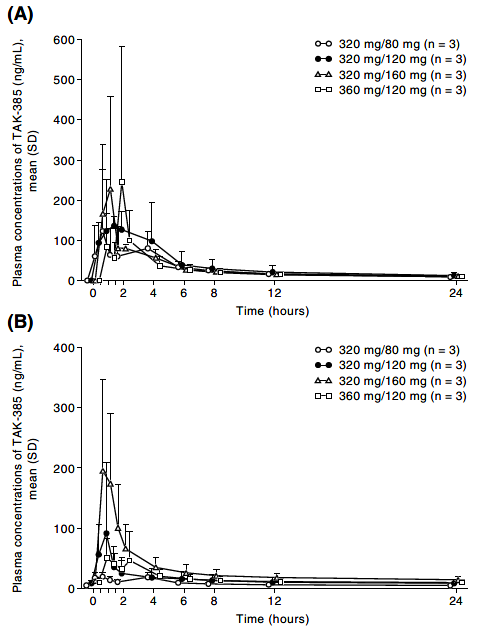


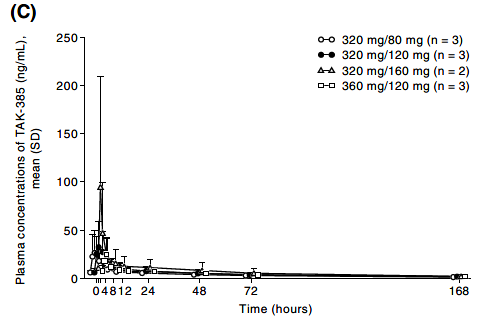


**SUPPLEMENTARY FIGURE S3** Mean (standard deviation) plasma concentration–time profiles of TAK-385 by dose group in the expansion phase (Part B). SD, standard deviation.


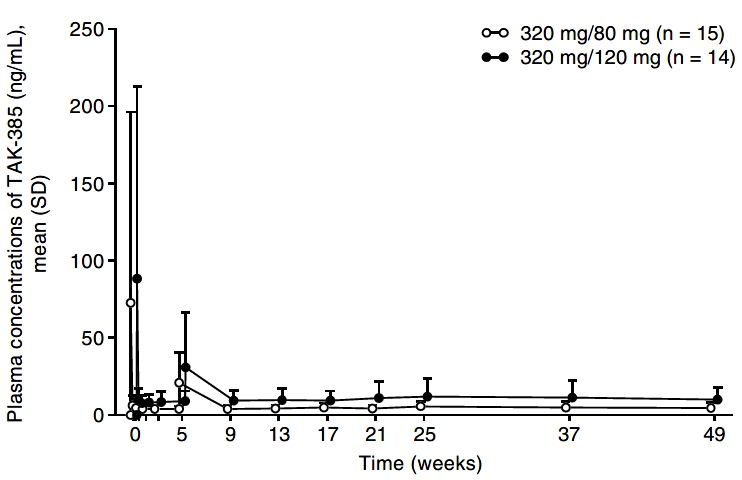


**SUPPLEMENTARY FIGURE S4** Mean (standard deviation) serum concentration–time profiles of luteinizing hormone, (A) by dose cohort in the dose-escalation phase (Part A), and (B) by dose group in the expansion phase (Part B). D, day; LH, luteinizing hormone; SD, standard deviation; WK, week.


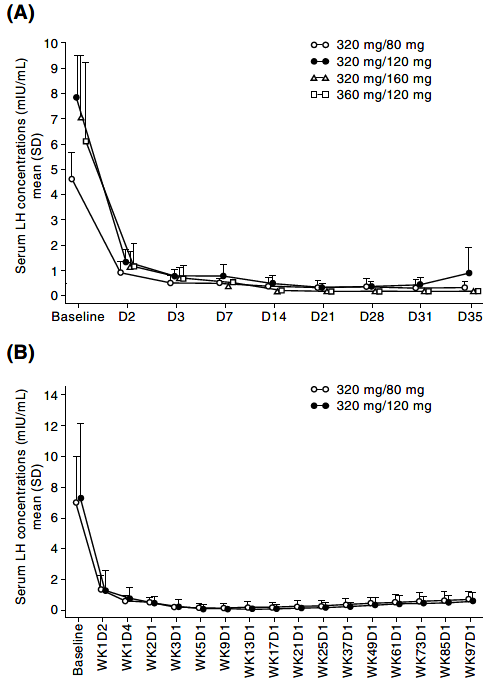


**SUPPLEMENTARY FIGURE S5** Mean (standard deviation) serum concentration–time profiles of follicle-stimulating hormone, (A) by dose cohort in the dose-escalation phase (Part A), and (B) by dose group in the expansion phase (Part B). D, day; FSH, follicle-stimulating hormone; SD, standard deviation; WK, week.


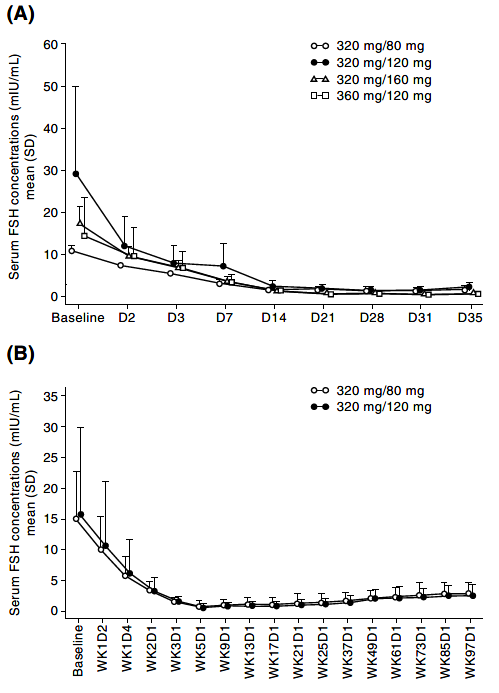

Supplement: Supplementary file 1 [file CAM4-8-5891-s001.docx]
